# Combination of Modified Bentall Procedure and Orthotopic Liver Transplantation

**DOI:** 10.1155/2018/6585879

**Published:** 2018-04-17

**Authors:** Philipp C. Seppelt, Rebecca von Haken, Jens Werner, Klaus Kallenbach

**Affiliations:** ^1^Department of Cardiology, Johann Wolfgang Goethe University Hospital, Frankfurt, Germany; ^2^Department of Anesthesiology, University Hospital Heidelberg, Heidelberg, Germany; ^3^Department of General and Visceral Surgery, Ludwig-Maximilians-University Hospital, Munich, Germany; ^4^Cardiac Surgery, INCCI HaerzZenter, Luxembourg, Luxembourg

## Abstract

Indication for combined cardiac surgery and orthotopic liver transplantation is rare and patients are at high risks. Individual surgical strategy must be developed since a general standard of such procedure does not exist. We report the case of a 45-year-old woman who underwent simultaneously modified Bentall procedure and orthotopic liver transplantation. Underlying diseases were end-stage polycystic liver, aneurysm of the ascending aorta, and severe aortic regurgitation. To avoid prolonged bypass times, both teams worked simultaneously. During cardiac reperfusion, time inferior vena cava stayed ligated while the cyst liver was explanted.

## 1. Introduction

Indication for concurrent cardiac surgery and orthotopic liver transplantation (OLTx) is rare and operation is not performed frequently due to challenging perioperative treatment. End-stage liver disease (ESLD) is associated with increased risk for bleeding and renal and liver failure and influences morbidity and mortality for patients undergoing cardiac surgery [1–3]. On the other hand, isolated OLTx in patients with uncorrected valvular heart disease is almost impossible. We report the case of a 45-year-old woman with ESLD and ascending aortic aneurysm with severe aortic valve insufficiency who underwent simultaneously modified Bentall procedure and OLTx.

## 2. Case Description

**Figure 1:**
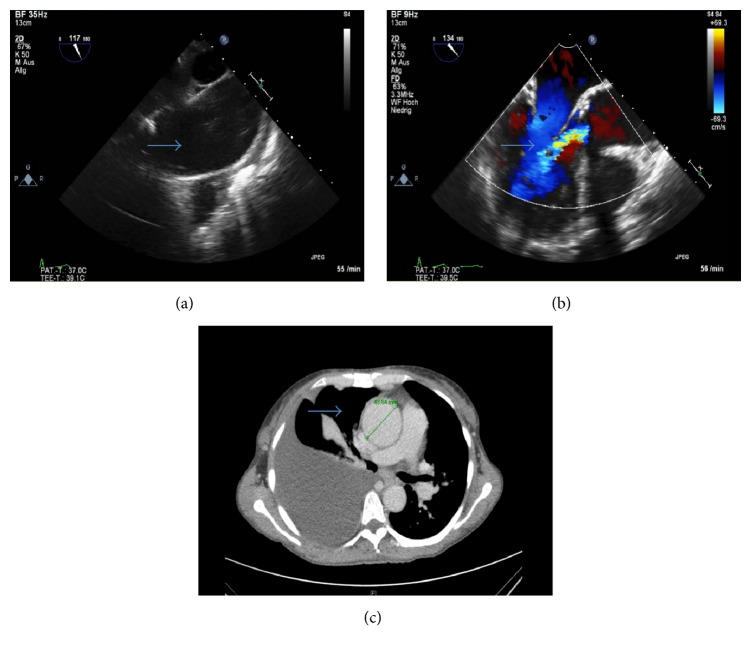
Aneurysm of ascending aorta ((a), blue arrow) and insufficiency of the aortic valve ((b), blue arrow, transesophageal echocardiography). Ascendens aneurysm was evaluated using CT-scan ((c), blue arrow, distal part of the aorta ascendens above the sinus valsalvae, early arterial phase).

**Figure 2:**
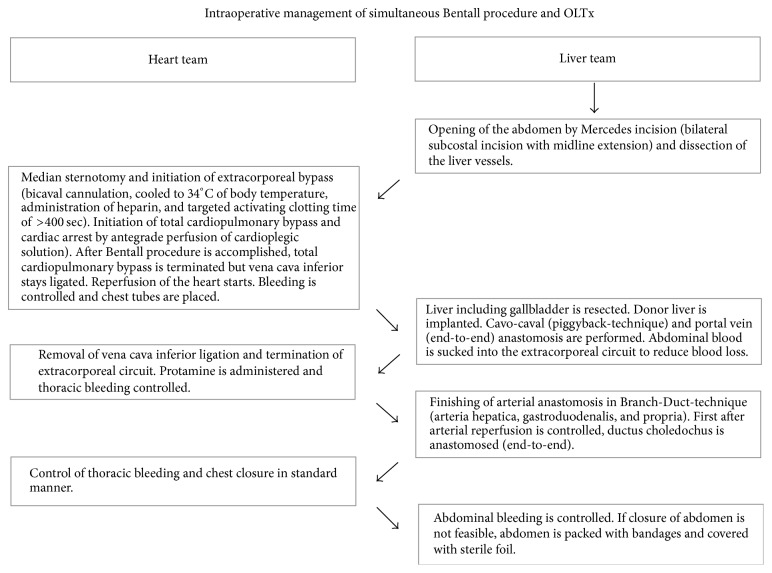
Surgical strategy of combined OLTx and Bentall operation. OLTx, orthotopic liver transplantation.

**Figure 3:**
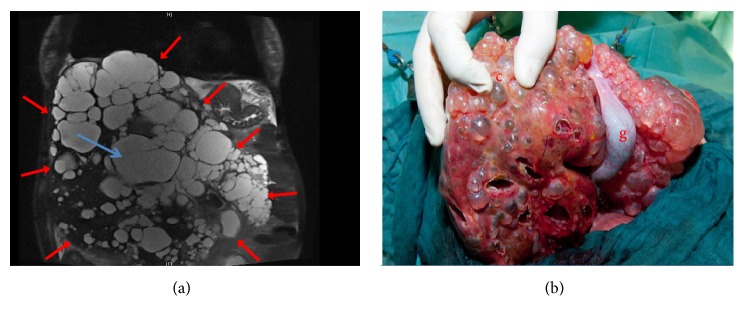
Well-perfused liver cysts (blue arrows) are highlighted by contrast medium (liver size marked with red arrows, preoperative computed tomography scan, early venous phase). Explanted cyst liver (b): gall bladder, g; cysts, c.

Underlying disease for liver transplant listing was polycystic liver disease (labMELD, model of end-stage liver disease 18.0, Child B). After several liver punctures, infected liver cysts (*Enterococcus faecalis* verified) drained continuously over a cystocutaneous fistula. During listing for liver transplantation, ascending aorta aneurysm (47 mm diameter) associated with severe insufficiency of bicuspid aortic valve was diagnosed (Figure 1). In order to avoid prolonged bypass and reperfusion times, decision was made against sequential cardiac surgery and OLTx. Instead, we chose a simultaneous surgical approach and both teams used the individual reperfusion times, respectively (Figure 2). The liver team started by bilateral subcostal incision with midline extension (Mercedes incision). The dissection of the liver vessels was troublesome and time-consuming due to severe intra-abdominal adhesions. Then heart team went on ECC via standard median sternotomy, bicaval cannulation, and cannulation of the distal aorta ascendens. Cardiac arrest was implemented by antegrade perfusion of 2000 ml Bretschneider solution and combined biological aortic valve and ascending aortic replacement as standard modified Bentall procedure was performed (27 mm Bio-Hancock II, T505, and 32 mm Hemashild Platinum prosthesis). Thoracic bleeding was controlled and chest tubes were placed. Due to unreconstructable bicuspid valve, a valve sparing technique, such as David operation, was not applicable. During cardiac reperfusion only inferior vena cava stayed ligated and the cyst liver was explanted in toto (Figure 3). After insertion of the transplant liver and cavo-caval and portal veins anastomosis by the abdominal team, cardiac surgeons removed the vena cava ligation and weaned ECC and Protamine was administered (130 min reperfusion time). First after the effect of Heparin was inhibited sufficiently, the arterial anastomoses were finished by the liver team in Branch-Duct-technique (arteria hepatica, gastroduodenalis, and propria). Arterial perfusion was controlled before ductus choledochus was finally anastomosed. Then, thoracic bleeding was controlled and chest closed in standard manner. As expected, abdominal hemostasis after ECG with systemic heparinization was troublesome due to diffuse intra-abdominal bleeding and a high amount of blood products was administered. The abdomen was let open but packed with bandages and covered with a sterile foil. The postoperative course was complicated due to two surgical revisions for abdominal hematoma. The patient was extubated successfully on the third postoperative day after final closure of the abdomen. Today, 748 days after liver transplantation, the patient is in good general conditions (NYHA I) with preserved function of the transplanted liver and good systolic function of the left ventricle (ejection fraction 55%).

## 3. Comment

Patients with ESLD and additional cardiovascular diseases, such as severe aortic regurgitation, do often not present with acceptable clinical conditions for OLTx. For these patients, a simultaneous procedure of heart surgery and liver transplantation is a rational approach [4]. Severe aortic regurgitation increases left ventricular preload causing increased myocardial wall stress and restricted coronary perfusion [5]. In our reported case, the presence of severe insufficiency of the aortic valve increased the risk for intraoperative acute cardiac decompensation with low cardiac output and was validated as absolute contraindication for sole OLTx. Because of infected and fast-growing liver cysts and severe coagulation disorders, a two-stage procedure with prior cardiac surgery and OLTx over the course of time was not a promising approach. The risk for life-threatening sepsis or bleeding complication after sole cardiac surgery was considered as too high. Although the risk for major abdominal bleeding complications is increased during extracorporeal circulation (ECC) requiring systemic heparinization (targeted activated clotting time > 400 sec), the risk of acute cardiac decompensation was considered as pivotal limitation for sole OLTx [6]. In our case, diffuse abdominal bleeding after weaning ECG was serious, potentially life-threatening, and very difficult to handle. However, we assume that a successful outcome in these patients is only achievable if the individual bypass times are kept as short as possible. Otherwise prognosis of these patients with coagulation disorders becomes infaust after surgery. In the presented case, vena cava stayed ligated exceptionally during cardiac reperfusion in order to facilitate appropriate conditions for the liver team and to reduce the operation time. We conclude that indication for simultaneous cardiac surgery and OLTx in patients with end-stage liver disease is seldom but feasible. Accurate strategy is necessary to reduce operation and bypass times, because bleeding complications are the major limitations of outcome in these high-risk patients.
